# A Model of the Home Literacy Environment and Family Risk of Reading Difficulty in Relation to Children’s Preschool Emergent Literacy

**DOI:** 10.1177/00222194231195623

**Published:** 2023-09-16

**Authors:** Sara Esmaeeli

**Affiliations:** 1 Department of Early Childhood Education, Faculty of Arts and Education,University of Stavanger, Norway; 2 Norwegian Centre for Reading, Education and Research, University of Stavanger, Norway

**Keywords:** home literacy environment (HLE), parents’ self-reported reading disability (RD), family risk of RD, preschool emergent literacy skills, multiple-deficit model of RD

## Abstract

This study extends the research on the preschool *home literacy environment* (HLE) in the context of the family risk (FR) of reading disability (RD) by examining a multiple-deficit model of RD. A total of 1,171 six-year-old Norwegian children were assessed at school entry, the onset of formal reading instruction in Norway. Their parents completed a questionnaire regarding their own RD, education, and the HLE. The final sample after applying the inclusion criteria was 794 children and their parents. The findings suggest, first, that two HLE factors (access to print and reading-related activities) should be distinguished rather than treated as a single factor (“exposure to print”) as the majority of previous studies have done. This finding suggests a three-factor HLE model that includes *parents’ reading interests and habits*, *reading-related activities*, and *access to print*. Second, family risk of RD is related to some extent to the HLE, even after controlling for parents’ education. Third, children’s experiences in their home environments and their emergent literacy may not be independent of their family risk of RD. More importantly, this study highlights the potential protective role of the HLE, especially when there is a history of RD within the family. The reason is that the positive association between the HLE and children’s code-related emergent literacy remains significant when controlling for family risk of RD (access to print → emergent literacy: 0.39 [0.01, 0.68], *p* < 0.01; reading-related activities → emergent literacy: 0.37 [0.02, 0.35], *p* < 0.01; parents’ reading interests and habits → emergent literacy: 0.26 [0.001, 0.15], *p* < 0.01). This finding supports that children’s emergent literacy can be improved via a modifiable, dynamic factor such as the HLE.

Emergent literacy skills, which include children’s preschool code- and meaning-related skills, refer to the preliteracy skills that form a foundation for the development of school literacy skills, including both reading and writing (Whitehurst & Lonigan, 2001). A meta-analysis reported that code-related preschool emergent literacy skills, such as letter knowledge and phonemic awareness, are good predictors of children’s later reading skills, while vocabulary is better at predicting reading comprehension (Lonigan et al., 2008). In addition, the process of building the foundation of emergent literacy involves exchanges between children and their environments (Whitehurst & Lonigan, 1998), particularly the *home literacy environment* (HLE). The HLE is therefore vital for the development of emergent literacy skills (Burgess et al., 2002; Rashid et al., 2005; Sénéchal & LeFevre, 2002, 2014).

## Family Risk of Reading Disabilities

In addition, the HLE is believed to be associated with family background, which includes not only parents’ levels of education and/or socioeconomic status (SES) (Carroll et al., 2019; Phillips & Lonigan, 2009) but also biological factors, as families share both genes and environments (Hart et al., 2009). In addition, research on the *family risk* of *reading disability* (FR of RD) has shown that when there is an incidence of RD within a family, the probability that a child will display emergent literacy difficulties may increase (Dilnot et al., 2017). In line with the research on FR of RD, some researchers (Carroll et al., 2014; Esmaeeli et al., 2019) have shown that parents’ self-reports of RD can also predict emergent literacy difficulties in their children (children with FR of RD or FR children).

The reason for such FR of RD is likely the shared genes and/or shared environments among family members or, more specifically, the complex interplay between these factors (Daucourt et al., 2020; Pennington, 2006). Accordingly, a multiple-deficit model is proposed for understanding developmental RD in a multilevel, and an interactive framework, including spanning genetic levels (e.g., FR of RD in this study), environmental factors and/or gene–environment interactions (e.g., the HLE) as well as cognitive levels, such as emergent literacy skills (McGrath et al., 2020; Pennington, 2006; van Bergen, de Jong, et al., 2014). The multiple-deficit model claims that RD is not a simple difficulty caused only by a single deficit at one of these three levels but that it arises from several deficits working at these interacting levels (e.g., genetics and cognitive or environmental factors).

Despite the large body of research that has focused on the HLE, FR of RD and their relationship with children’s emergent literacy, several questions remain unanswered, as discussed in this multiple-deficit model of RD.

## Models of Home Literacy Environments

The HLE is typically measured using rating scales via parent questionnaires, but there is a great deal of variety in both the measures and the factor structure that have been used to investigate the HLE. The earliest research has mostly measured the HLE in terms of the frequency of *shared reading activities* at home. However, this HLE model has been criticized for being methodologically limited (Burgess et al., 2002; Lonigan, 1994) because it considers only one aspect of the HLE and because the studies investigating this model have used small sample sizes.

Sénéchal et al. (1998) were the first to introduce two different aspects of the HLE: *formal* and *informal*. A formal HLE typically refers to code-based activities in which parents explicitly teach the letters, sounds, reading, or spelling of some words. In contrast, an informal HLE refers to reading-related activities that focus on reading a book with/for the child. Such an informal HLE consists mostly of meaning-based interactions relating to the concept of the story, which can enhance the word knowledge of children.

A common measure of an *informal* HLE is *storybook exposure*, which consists of *shared reading* (such as parent–child reading-related activities at home and the age at which parents start reading to their children, i.e., the onset of shared reading activities) and *access to print* (i.e., the variety of print material in the home, such as the number of children’s books and library visits with the child). Sénéchal (2006), for example, reported that the aspect of *parents’ teaching* directly predicted children’s letter knowledge in kindergarten and reading fluency in the fourth grade, while *storybook exposure* predicted children’s kindergarten vocabulary directly and predicted their fourth-grade reading comprehension indirectly.

Burgess et al. (2002) defined the HLE as consisting of a variety of reading-related components, including various attitudes, resources, and activities in the home. Accordingly, the HLE was subdivided into *active* and *passive* aspects. To some extent, this distinction resembles that of the *formal* and *informal* HLE, which was introduced earlier by Sénéchal et al. (1998). However, the *active* and *passive* HLE distinction does not include the formal aspect of the HLE (i.e., parents’ teaching). The active HLE includes activities in which parents engage the child directly in shared reading and reading-related activities, such as shared book reading. The passive HLE includes the opportunities that parents provide for their children to encounter the world of literacy in different ways, for example, by providing children’s books in the household and/or access to a library or by serving as role models for their children in regard to reading books as a pleasure activity. The passive HLE indirectly helps children enrich their knowledge, skills, and their literacy interest through the books and other print materials that are available in the home and by observing and experiencing the parents’ appreciation of and engagement in reading-related activities. Burgess et al. (2002) found that the active HLE was a stronger contributor to children’s emergent literacy and word-reading skills than the passive HLE. However, their description of the passive HLE included only the parents’ own reading interests and habits and the frequency with which the children watched TV. These authors did not include *access to print* domains, such as *the number of children’s books in the household*, which, according to their definition, could be included in the passive HLE. This limitation might explain the nonsignificant results for the association between the passive HLE and children’s emergent literacy in Burgess’s findings. In contrast, Weigel et al. (2006) divided the HLE into two dimensions: *parents’ reading interests* and *storybook exposure*. Weigel et al. (2006) found that the parents’ reading interest was the foundation for the HLE, as this dimension was associated with storybook exposure and directly predicted both the children’s emergent writing skills and their receptive oral language skills.

Sénéchal et al. (1998) defined the *informal* HLE based on the unitary construct of *exposure to print*, which consists of two domains: *access to print* and *reading-related activities* in the home. However, in view of their definition of *active and passive* HLE, one may conclude that Sénéchal’s *exposure to print* should not be considered to constitute a unitary construct but rather a two-dimensional construct comprising two distinct factors that should not have been united under a single construct: (a) *access to print*, which can be considered a passive aspect of the HLE, and (b) *reading-related activities*, which can be considered an active aspect of the HLE.

**Figure 1.**
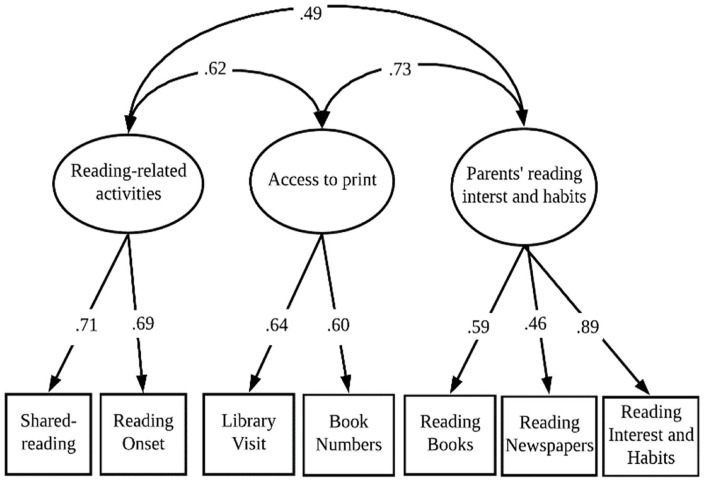
Model 1. A Three-Factor Model of the Home Literacy Environment *Note*. → Significant pathway.

In addition, a broad HLE perspective—which includes the three aspects of *reading-related activities*, *access to print*, and *parents’ reading interests and habits*—has been suggested in the literature (Esmaeeli et al., 2018, 2019; Torppa et al., 2007). However, these previous studies have not empirically investigated this three-factor HLE model. The present study has three aims, and the first one is to investigate the three-factor HLE model (see Figure 1), which consists of *reading-related activities*, *access to print*, and *parents’ reading interests and habits*.

### Home Literacy as an Early Intervention Program

Home literacy intervention programs provide an effective context in which parents can promote their children’s early emergent literacy development. Over the years, various home/family literacy programs have been developed to promote children’s emergent literacy, and their subsequent literacy skills. Consequently, a number of meta-analyses have attempted to summarize the effectiveness of these home literacy programs, which has resulted in different mean effects ranging from low to high (see Fikrat-Wevers et al., 2021, for the meta-analysis). The meta-analysis conducted by Fikrat-Wevers revealed that home literacy programs could enhance children’s emergent literacy and their subsequent literacy skills. More importantly, home literacy programs can be beneficial even for children from families with a low SES. This meta-analysis confirmed that introducing home literacy activities to parents who are not familiar with such activities may generally improve the HLE, children’s emergent literacy (*d* = 0.50) and their subsequent literacy skills (*d* = 0.16).

In another meta-analysis, various activities were found to be characteristics of an effective home literacy program. In this meta-analysis, Flack et al. (2018) found that *how* stories were read (reading style) was more important than *who* read them. For example, dialogic reading styles that encourage additional interactions with the text—such as text-related talk, using open-ended questions, repetition, and picture-pointing methods during shared reading activities—have been recommended as effective features for an effective home literacy program.

### Home Literacy Environment and Family Risk of Reading Disabilities

The HLE has been compared between families with and without FR of RD, but the data that have been found in this context have been limited and have resulted in mixed findings (Esmaeeli et al., 2018; Hamilton et al., 2016). Several studies have found that neither shared reading activities nor access to print differed between families with and without FR of RD, despite the fact that parents in the FR group (vs. the group without FR of RD) scored lower on measure of *parents’ reading interests and habits* (Lyytinen et al., 2004; Torppa et al., 2007).

**Figure 2.**
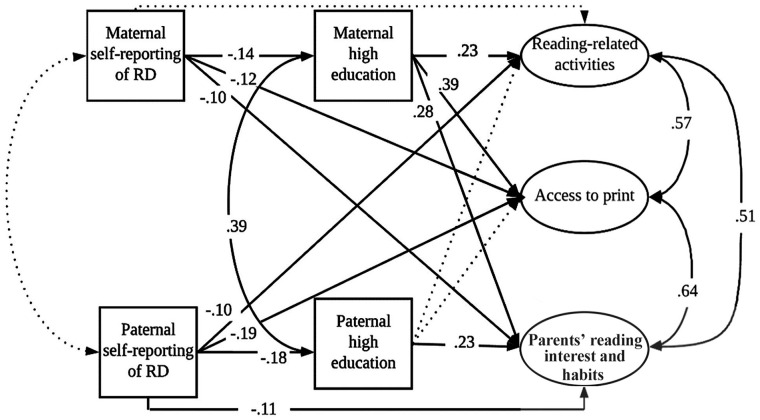
Model 2. Parents’ Self-Reported RD and Their Level of Education in Relation to Three Domains of the Home Literacy Environment. *Note. →* Significant pathway. 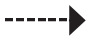 Nonsignificant pathway. RD = reading disability.

In contrast, some other studies have found differences in both *shared reading activities* and *access to print* between families with and without FR of RD (Dilnot et al., 2017; Esmaeeli et al., 2018; Hamilton et al., 2016). For example, Dilnot et al. (2017) found that children with (vs. without) FR of RD experienced more environmental disadvantages in terms of both their family’s SES (including their parents’ levels of education and occupations) and the HLE. Hamilton et al. (2016) also reported group differences in the frequency of shared reading and the number of children’s books in the household between families with and without FR of RD; however, when the authors controlled for family SES, the statistically significant group differences disappeared. Similarly, Esmaeeli et al. (2018) found that families without parents with self-reported RD reported a richer literacy environment for the children in terms of all three HLE aspects (*parents’ reading interests and habits*, *reading-related activities*, and *access to print*) than families in which at least one parent self-reported RD. Esmaeeli et al. (2018) suggested that the difference in parents’ educational levels could be a possible explanation for group differences in the HLE between families with and without FR of RD. However, less is known regarding the association between the HLE and FR of RD after accounting for parents’ educational levels. The second aim of this study is to investigate the association between the HLE and FR of RD (indexed through maternal and paternal self-reported RD) while accounting for parents’ educational levels. This complex, interactive model (see Figure 2) allows us to study how maternal and paternal self-reported RD are linked to each domain of the HLE (*parents’ reading interests and habits, reading-related activities*, and *access to print*) while accounting for parents’ educational levels.

### Home Literacy Environment, Family Risk of Reading Disabilities and Children’s Emergent Literacy

In a meta-analysis of the research on FR of RD, Snowling and Melby-Lervåg (2016) reported that universally and regardless of language, children with FR of RD performed poorly in terms of emergent literacy. A growing body of research has addressed the association between the HLE, children’s emergent literacy and FR of RD based on a multiple-deficit model of RD (e.g., Khanolainen et al., 2020, 2023; Salminen et al., 2021; Silinskas et al., 2020; Torppa et al., 2022). For example, Torppa et al. (2022) found a longstanding effect of the HLE on children’s later reading comprehension that started from a very early age (i.e., 2 years), while FR of RD was included as a moderator. Few studies, however, have addressed the association between the HLE and children’s emergent literacy while accounting for the parents’ educational levels and FR of RD in a multiple-deficit model of RD before the onset of formal reading instruction or formal schooling.

Formal reading instruction may impact children’s emergent literacy skills (e.g., their letter knowledge and phonemic awareness). Crone and Whitehurst (1999) found that children who began school a year earlier than same-age peers outperformed these peers on measures of both emergent literacy skills and early reading skills. Therefore, it is important to investigate a concurrent, multiple-deficit model of RD prior to the onset of formal schooling, as is the focus of the present study. In the present study, children’s emergent literacy skills were assessed at the onset of formal reading instruction, that is, at the onset of formal schooling. This approach allows us to control for the association between formal reading instruction and children’s preschool code-related emergent literacy skills.

**Figure 3.**
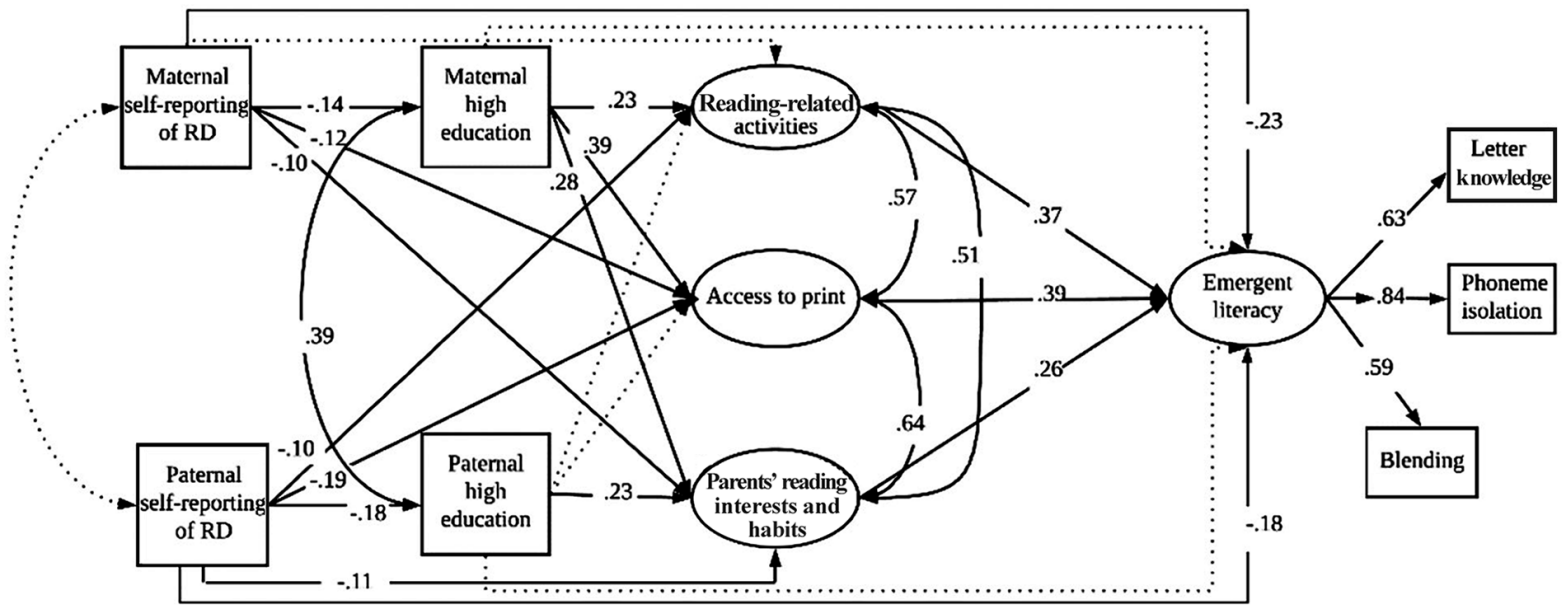
Model 3. Parents’ Self-Reported RD and Their Level of Education in Relation to Three Domains of the Home Literacy Environment, and Children’s Emergent Literacy. *Note. →* Significant pathway 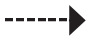 Nonsignificant pathway. RD = reading disability.

The third and final aim of this study is to investigate the association between the HLE and children’s code-related preschool emergent literacy while concurrently accounting for the parents’ educational levels and FR of RD (controlling for the mothers’ and fathers’ educational levels and their FR of RD separately and simultaneously). Code-related preschool emergent literacy skills, such as letter knowledge and phonemic awareness, are known to be better predictors of children’s reading outcomes than other emergent literacy skills, such as vocabulary, which is the best predictor of reading comprehension (Lonigan et al., 2008; Niklas & Schneider, 2013). This multiple-deficit model of RD (see Figure 3) may contribute to the literature by showing how FR of RD is linked with parents’ educational levels, each domain of the informal HLE (*parents’ reading interests and habits, reading-related activities*, and *access to print*), and the child’s emergent literacy skills in a concurrent association model. Within the multiple-deficit model of RD, a risk factor such as FR of RD may increase the likelihood of deficits in the emergent literacy of children with FR of RD. However, environmental factors may act as either risk or protective factors (Pennington, 2006; van Bergen, van der Leij, & de Jong, 2014).

In summary, this study aims to answer the following research questions:

**Research Question 1:** Can HLE be described by a model consisting of three factors of *access to print* and *reading-related activities*, in addition to *parents’ reading interests and habits* (see Figure 1)?

It is expected for the HLE model to consist of three factors, in which the two factors of *access to print* and *reading-related activities* can be distinguished from one another rather than treated as a single factor known as *“exposure to print,”* as the majority of previous studies have used.

**Research Question 2:** Is FR of RD associated with HLE while controlling for parents’ educational level (see Figure 2)?

It is expected that FR of RD to be partially associated with the HLE even after accounting for parents’ educational level. Previous research (Dilnot et al., 2017; Esmaeeli et al., 2018; Hamilton et al., 2016) has shown that children from families without (vs. with) FR of RD usually experience richer home literacy.

**Research Question 3:** What is the contribution of the HLE to children’s code-related emergent literacy skills in preschool after accounting for the role of FR of RD as a risk factor (see Figure 3)?

The HLE is expected to be positively associated with children’s preschool outcomes in code-related emergent literacy after accounting for FR and the parents’ educational level. In this multiple-deficit model of RD (see Figure 3), HLE and parents’ educational level are included as possible protective factors at the environmental level and/or at the level of gene–environment interaction. However, code-related emergent literacy is discussed at the cognitive level, and FR of RD is used as a proxy for a genetic risk factor (van Bergen, van der Leij, & de Jong, 2014).

## Method

### Context of the Study

The participants in this study were selected from a longitudinal project (*On Trac*). Project *On Track* recruited a convenience sample (Lundetræ et al., 2017) including 1,171 children from 16 primary schools at school entry (the beginning of first grade), which is the onset of formal reading instruction in Norway. Norwegian Early Childhood Education and Care (ECEC) includes the age group of 0- to 6-year-old children, and all children start formal reading instruction in the calendar year in which they turn 6 years old, which is also when they start first grade of formal schooling. Norwegian ECEC offers a framework but not an official curriculum for prereading, reading-related activities, or reading instruction. The framework only encourages ECEC teachers to practice informal reading-related activities such as shared reading; however, the formal teaching of the alphabet and the letter sounds may begin in the first grade of the primary school when formal schooling begins. The participants in this study were 6-year-old children from On Track project at school entry who were tested at the beginning of the first grade before formal reading instruction had begun and could possibly impact their preschool emergent literacy skills.

### Procedures, Participants, and Measures

A team of 18 trained testers administered the test battery individually to the participating children using a digital tablet at the beginning of the first grade at their school in a quiet classroom. Norwegian primary schools usually invite parents to attend a welcome meeting before their children begin formal schooling for the first time. These meetings held by the participating schools gave our research group the opportunity to attend and gave parents both written (leaflet) and oral presentations about the project and invited them to take part in the study.

The parents in the participating schools received a parental consent form and a questionnaire to be completed later at home. The parents’ questionnaire included questions regarding demographic information, the HLE, FR of RD, and the child’s language background. All the measures used in this study were constructed by the research group; the validity and reliability of these measures have been tested previously.

This study excluded children who were second-language speakers or had hearing problems or other known disabilities, or who had participated in any language or literacy intervention program (*n =* 317) according to their parent’s report; children whose parents did not consent to their participation (*n =* 28); and children whose parents did not provide information regarding their RD status (*n =* 20) or who answered “I don’t know” in response to this question (*n =* 12). After applying the exclusion criteria, the final sample consisted of 794 children (*mean age =* 6.22, *SD =* 0.28; *boys =* 48.5%). Of this final sample, 9.76% were reported as single-parent children, who lived mostly with their mother (91.6%).

#### Parents’ Self-Reports of RD as an Index for FR of RD

FR of RD was indexed by parents’ self-reports of RD, as has been done in some previous studies (Carroll et al., 2014; Esmaeeli et al., 2019). This measure of the parents’ RD status was obtained from the parent questionnaire through two separate questions asked about the child’s biological parents: “Has the child’s biological mother had ‘*reading and writing disability*’?” The response options were “Yes,” “No,” and “I don’t know.” The same question and response options asked about child’s biological father.

“*Reading and writing disability*” was a familiar term among the individuals in this research sample, as it is frequently used by schools and practitioners who work in the RD field; RD was defined as “*difficulties* with word recognition and spelling.” The term “*reading and writing disability*” was also discussed as word recognition and spelling disabilities at the welcome meeting.

From the whole sample, 160 children (20.1%; *n* = 794) had a parent with a self-reported RD and were categorized as children with FR of RD in this study.

#### Parents’ Educational Levels

A measure of the parents’ educational levels was obtained via two separate questions: one for the mother and one for the father. The question consisted of multiple choices: no “education” (0%), “elementary school” (0%), “lower secondary school” (4.4%), “diploma from upper secondary school” (27.2%), and “college/university degree” (68.4%). In this sample, parents unsurprisingly reported mostly high levels of education, since elementary and lower secondary schools are mandatory in (country name omitted). For the purpose of this study, the parents who had completed a college/university degree were considered to have a high level of education, while those with a diploma from upper secondary school or less were considered to have a low level of education (see Table 1).

**Table 1.** Parents’ Education and the HLE Measures: Descriptive Statistics for the Whole Sample, Children With and Without FR, and the Group Comparison of Means With Effect Sizes

	Whole sample				Children without FR				Children with FR				Mann–Whitney *U/t* test (*df*)	Cohen’s *d*^a^
Variables	*N*	*M* (*SD*)/%	Minimum	Maximum	*n*	*M* (*SD*)/%	Minimum	Maximum	*n*	*M* (*SD*)/%	Minimum	Maximum		
Living status of the child (parents live together, %)	794	84.8%	—	—	634	85.7%	—	—	160	84.2%	—	—	—	—
Live mostly with mother if the parents are not living together	794	92%	—	—	634	99.9%	—	—	160	97.1%	—	—	—	—
Years in kindergarten	794	4.58 (0.77)	1	5	634	4.62 (0.71)	1	5	160	4.45 (0.94)	1	5	—	—
Child’s mother answered the questionnaire (%)	494	62.2%	—	—	392	61.8%	—	—	102	63.8%	—	—	—	—
Maternal education^b^	794	0.69 (0.46)	0	1	634	0.74 (0.44)	0	1	160	0.53 (0.50)	0	1	*Mann–Whitney U*: 35,388.00***	.43
Paternal education^b^	794	0.57 (0.50)	0	1	634	0.62 (0.49)	0	1	160	0.36 (0.48)	0	1	*Mann–Whitney U* 32,106.00***	.53
Home Literacy Environment (HLE)														
Children’s books in home	794	4.55 (0.74)	1	5	634	4.61 (0.68)	2	5	160	4.32 (0.91)	1	5	*Mann–Whitney U* 42,394.50***	.23
Onset of shared reading	794	4.84 (0.45)	2	5	634	4.85 (0.43)	2	5	160	4.77 (0.50)	2	5	*Mann–Whitney U* 46,649.00***	.11
Frequency of shared reading	793	4.05 (0.96)	1	5	633	4.09 (0.94)	2	5	160	3.90 (1.02)	1	5	*Mann–Whitney U* 45,169.50*	.15
Frequency of library visits with child	788	3.48 (0.75)	1	4	630	3.49 (0.73)	1	4	160	3.43 (0.82)	1	4	*Mann–Whitney U* 48,468.00**	.06
Parents own book reading	790	3.34 (1.41)	1	5	631	3.40 (1.39)	1	5	159	3.09 (1.47)	1	5	*Mann–Whitney U* 43,976.00**	.19
Parents reading newspapers and magazines	793	4.79 (0.63)	1	5	634	4.83 (0.55)	1	5	159	4.62 (0.87)	1	5	*Mann–Whitney U* 45,909.50***	.13
Parents’ interests in reading	790	3.70 (0.67)	1	4	632	3.74 (0.61)	1	4	158	3.54 (0.85)	1	4	*Mann–Whitney U* 41,957.50***	.24
Emergent literacy														
Letter knowledge	794	12.20 (3.30)	0	15	634	12.50 (3.21)	0	15	160	11.01 (3.41)	2	15	*t test*: 234.79 = 5.79***	.51
First-phoneme isolation	794	5.61 (2.89)	0	8	634	5.97 (2.70)	0	8	160	4.18 (3.13)	0	8	*t test*: 222.37 = 6.89***	.61
Blending	794	3.68 (2.65)	0	8	634	3.90 (2.66)	0	8	160	2.81 (2.42)	0	8	*t test*: 264.20 = 4.98***	.44

*Note.* FR = family risk.

a *Cohen’s d* (Lenhard & Lenhard, 2016). ^b^Parents’ level of education: 0 = secondary school; 1 = university/college.

* *p* < 0.05. **p < 0.01. ***p < 0.001.

#### Home Literacy Environment

The measure of home literacy was developed based on previous research (e.g., Burgess et al., 2002; Niklas & Schneider, 2013; Torppa et al., 2007) and included in the parent questionnaire. This HLE questionnaire includes different items regarding reading-related activities such as frequency of parent–child shared reading, parents’ providing of reading materials for their children, and parents’ own reading interests and habits. More detailed information regarding this HLE questionnaire is included in the online supplemental materials appendix.

#### Code-Related Emergent Literacy Skills

##### Letter Knowledge

This task consisted of 15 multiple-choice items. For each item, the child was asked to listen to a prerecorded letter sound on the tablet and then to respond by pressing one of four letters shown on a touch screen (Cronbach’s α = 0.85).

##### First-Phoneme Isolation

This task consisted of eight items. For each item, an object was first shown on the tablet screen. Then, the examiner named the object while pointing at it. The child was then asked to produce the first sound in the word. The examiner scored the child’s response directly on the tablet. The task was automatically discontinued if a child failed two subsequent items (Cronbach’s α = 0.92).

##### Blending Task

This task consisted of eight items with increasing difficulty. For each item, a set of phonemes forming a word was presented orally by the examiner in the correct order but pronounced separately. Then, the child was asked to “blend” the phonemes, that is, to put them together to form the corresponding word. The task was automatically discontinued if a child failed two subsequent items (Cronbach’s α = 0.86).

A construct of code-related emergent literacy was designed through a confirmatory factor analysis (*CFA*) using a least squares estimator (*WLSMV*). The results showed a good model fit, *χ*^2^(374) = 622.03, *p* < 0.001; root mean square error of approximation (*RMSEA*) = 0.29, 90% confidence interval (*CI*) = [0.025–0.033]; comparative fit index (*CFI*) = 0.99; Tucker–Lewis’s index (*TLI*) = 0.99); the indicators were strongly related to the construct of emergent literacy.

### Statistical Analysis

The number of missing values across the variables for the whole dataset was less than 0.5%. The values for both skewness and kurtosis with regard to all the variables were ± 2. The measurement modeling was conducted in Mplus 8 using a least squares estimator (WLSMV), which is a robust estimator that does not assume a normal distribution and provides the best option for modeling categorical or ordered data (Muthén & Muthén, 2017). The model fit was assessed with the chi-square statistic, *CFI, TLI*, and *RMSEA*. For a well-fitting model, *CFI* and *TLI* values equal to or greater than 0.95 and *RMSEA* values equal to or below 0.05 are preferred (Byrne, 2013).

The data were structured hierarchically, as the estimated intraclass correlation (*ICC*) analyses indicated that class membership at the school level did not account for a substantial portion of the individual differences in any of the code-related emergent literacy scores: letter knowledge (0.029), first-phoneme isolation (0.05), and blending (0.02).

## Results

### Descriptive Statistics

Table 1 shows the descriptive statistics of the maternal and paternal educational levels, the HLE components, and children’s preschool code-related emergent literacy for the whole sample and for the groups of children with and without FR of RD as well as the results of the significance tests of the differences between these two groups.

Both the mothers (*d* = 0.45) and the fathers (*d* = 0.54) in the group without FR of RD reported higher educational levels than did those in the FR group. In addition, participants in the group without FR of RD scored significantly higher on all of the HLE components. Regarding code-related emergent literacy, the children with FR of RD performed significantly poorer than those without FR of RD on all three components: letter knowledge (*d* = 0.45), first-phoneme isolation (*d =* 0.61), and blending (*d =* 0.43).

### Correlations

The correlations between the latent variables were calculated in Mplus and are shown in Table 2. The results showed that all three factors of the HLE correlated negatively with both maternal RD and paternal RD. As expected, all three HLE factors were also positively correlated with the maternal and paternal educational levels.

**Table 2.** Correlation Between Measures and Latent Factors for the Whole Sample.

	1.	2.	3.	4.	5.	6.	7.
1. Maternal self-reporting of RD^a^							
2. Paternal self-reporting of RD^a^	0.17*						
3. Maternal education	−0.23*	−0.19*					
4. Paternal education	−0.12*	−0.30*	0.41*				
5. Access to print^b^	−0.33*	−0.35*	0.42*	0.24*			
6. Reading-related activities^b^	−0.03	−0.20*	0.21*	0.14*	0.61*		
7. Parents’ literacy interests and habits^b^	−0.26*	−0.26*	0.36*	0.25*	0.71*	0.54*	
8. Emergent literacy^b^	−0.41*	−0.31*	0.27*	0.20*	0.42*	0.33*	0.30*

a RD = reading difficulties. ^b^Latent Score.

* *p* < 0.001.

Children’s preschool code-related emergent literacy was negatively correlated with maternal RD (−0.41) and paternal RD (−0.31) but positively correlated with both the maternal (0.27) and paternal educational levels (0.20) and with the three HLE factors: *access to print* (0.42), *reading-related activities* (0.33), and *parents’ reading interests and habits* (0.30).

### Three-Factor Home Literacy Model

To address the first aim, a series of CFAs were run to test the best-fitting HLE model. First, a single-factor HLE model was defined that included all the HLE components. Second, a two-factor model was designed based on a model suggested by Burgess et al. (2002), where the factors were (a) active HLE—library visits, shared reading, and the frequency of watching TV and playing digital games—and (b) passive HLE—the number of children’s books in the home, onset of shared reading and parents’ own interests and reading habits. For these two initial models, two of the HLE components (frequency of playing digital games and frequency of watching TV) were found to load significantly but weakly onto the related latent variable. Hence, these two components were excluded from the further analysis. The fitting criterion showed that neither the single-factor model, *χ*^2^(14) = 151.88 (*p* < 0.001); *RMSEA* = 0.112, 90% CI = [0.096–0.128]; *CFI* = 0.897; *TLI* = 0.846, nor the two-factor model, *χ*^2^(13) = 101.10 (*p* < 0.001); *RMSEA* = 0.093, 90% *CI* = [0.076–0.110]; *CFI* = 0.934; *TLI* = 0.894, adequately fit the data.

After examining these first two models, four alternative models were considered: a three-factor model, a second-order three-factor model, a three-factor bifactor model, and a four-factor model. Among these alternative models, only the three-factor model including parents’ reading interests and habits, reading-related activities, and access to print, showed adequate fit to the data, while the other models were not identified. Figure 1 presents this three-factor model of the HLE that fit the data adequately (*RMSEA = 0.020, 90% CI =* [*0.000–0.0045*]; *CFI = 0.996; TLI = 0.993)*.

The participants’ mothers and fathers were free to choose which of them would answer the HLE questionnaire. In most cases, the questionnaire was answered by the child’s mother: the respondent was the mother for 61.8% of the children whose parents self-reported RD and for 63.8% of children whose parents did not self-report RD (see Table 1). To examine whether the respondent identity affected the results, the three-factor HLE model was also tested on a separate dataset including data from questionnaires answered only by the mothers (*n* = 498). This analysis indicated no significant changes to either the relationship pattern or the model fit, *χ*^2^(11) = 13.05 (*p* = 0.29); *RMSEA* = 0.020, 90% *CI* = [0.000–0.054]; *CFI* = 0.997; *TLI* = 0.995. These results suggest that this three-factor HLE model could be used in the whole sample to test the second aim of the study.

### Associations Between the Home Literacy Environment, Parents’ Self-Reported RD, and Parents’ Educational Levels

To address the second aim of this study, the three-factor HLE model was used to identify the associations between each HLE domain and parents’ self-reported RD while accounting for the parents’ educational levels. The mediating pathway of the parents’ educational levels were also tested using the bootstrapping method (Muthén & Muthén, 2017).

As shown in Figure 2, maternal and paternal self-reported RD were added as direct predictors of the three HLE domains and indirect predictors via the high maternal and paternal educational levels. The model (Model 2) showed a good fit to the *data*, *χ^2^*(39) = 90.46 (*p* < 0.001); *RMSEA* = 0.041, 90% *CI* = [0.030–0.052]; *CFI* = 0.961; *TLI* = 0.946. As seen in Figure 2, unsurprisingly, a negative and significant association was found between maternal self-reported RD and the maternal educational level as well as between paternal self-reported RD and the paternal educational level. Of all the HLE domains, *access to print* and *parents’ reading interests and habits* were negatively and directly associated with both maternal and paternal self-reported RD. In addition, the direct path was significant from paternal RD to the domain of *reading-related activities*, but there was no direct, significant link between maternal RD and this HLE domain. The associations between the maternal educational level and each HLE domain were also positive and significant, whereas the paternal educational level was significantly associated only with *parents’ reading interests and habits*.

Regarding the indirect associations (parents’ self-reported RD → parents’ educational levels → domains of the HLE), maternal self-reported RD was indirectly and negatively associated with all the HLE domains. However, paternal self-reported RD was indirectly associated only with *parents’ reading interests and habits* (see Table 3).

**Table 3.** Direct and Indirect Associations Between Family Risk Status, Parents’ Educational Level, and HLE.

Model 2	Standardized estimate	95% CI	*p* value
Maternal RD → access to print	−0.12	[−0.20, −0.03]	0.001
Paternal RD → access to print	−0.19	[−0.32, −0.10]	0.001
Maternal RD → reading-related activities	0.003	[−0.16, 0.001]	0.46
Paternal RD → reading-related activities	−0.10	[−0.16, −0.01]	0.05
Maternal RD → parents’ reading interests and habits	−0.10	[−0.17, 0.01]	0.05
Paternal RD → parents’ reading interests and habits	−0.11	[−0.17, 0.01]	0.05
Maternal RD → education	−0.14	[−0.22, −0.07]	0.001
Paternal RD → education	−0.18	[−0.26, −0.11]	0.001
Maternal RD → education → access to print	−0.06	[−0.08, −0.02]	0.001
Paternal RD → education → access to print	−0.01	[−0.04, 0.01]	0.29
Maternal RD → education → reading-related activities	−0.03	[−0.06, −0.02]	0.001
Paternal RD → education → reading-related activities	−0.002	[−0.05, 0.00]	0.18
Maternal RD → education → parents’ reading interests and habits	−0.04	[−0.08, −0.02]	0.001
Paternal RD → education → parents’ reading interests and habits	−0.02	[−0.07, −0.02]	0.01
Maternal education → access to print	0.39	[0.27, 0.51]	0.001
Paternal education → access to print	0.08	[0.04, 0.45]	0.23
Maternal education → reading-related activities	0.23	[0.15, 0.31]	0.001
Paternal education → reading-related activities	0.10	[0.001, 0.20]	0.09
Maternal education → parents’ reading interests and habits	0.28	[0.19, 0.41]	0.001
Paternal education → parents’ reading interests and habits	0.23	[0.20, 0.41]	0.001

*Note.* CI = confidence interval; RD = reading disability.

A Wald test was applied to examine whether the direct and indirect pathways from maternal RD to each of the three domains of the HLE differed significantly from those of paternal pathways. The results showed no significant differences, *χ^2^*(4) *=* 3.84, *p* = .43. This finding suggests that the associations between parents’ self-reported RD and the HLE domains did not differ statistically between the mothers and the fathers when their educational levels were accounted for.

### Home Literacy Environment and Children’s Preschool Code-Related Emergent Literacy

The third and final aim of the present study was to investigate the association between the HLE and children’s code-related emergent literacy and parents’ self-reported RD while accounting for the parents’ educational levels. To address this aim, maternal and paternal self-reported RD, the parents’ educational levels, and the three HLE domains were added as the direct and indirect predictors of children’s code-related emergent literacy (see Figure 3). The model fit the data adequately, *χ^2^*(760) = 1065.50 (*p* = .12); *RMSEA* = 0.023, 90% CI = [0.020–0.026]; *CFI* = 0.992; *TLI* = 0.991.

This figure (Model 3) indicates that both maternal and paternal self-reported RD were directly and negatively associated with children’s code-related emergent literacy, while positive links were found among all three domains of the HLE and children’s code-related emergent literacy. In this model, none of the indirect paths was significant from parents’ self-reported RD to children’s code-related emergent literacy (see Table 4).

**Table 4.** Direct and Indirect Associations Between Family Risk Status, Parents’ Educational Level, HLE, and Children’s Outcomes in Emergent Literacy at the Onset of Formal Reading Instruction.

Model 3	Standardized estimate	95% CI	*p* value
Maternal RD → emergent literacy	−0.23	[−0.29, −0.12]	0.001
Paternal RD → emergent literacy	−0.18	[−0.19, −0.02]	0.001
Maternal RD → education	−0.14	[–0.23, –0.07]	0.001
Paternal RD → education	−0.18	[−0.23, −0.10]	0.01
Maternal RD → education → access to print → emergent literacy	−0.02	[−0.05, −0.002]	0.23
Paternal RD → education → access to print → emergent literacy	−0.004	[−0.001, 0.03]	0.34
Maternal RD → education → reading-related activities → emergent literacy	−0.01	[−0.02, −0.001]	0.10
Paternal RD → education → reading-related activities → emergent literacy	−0.001	[−0.001, 0.002]	0.69
Maternal RD → education → parents’ reading interests and habits → emergent literacy	−0.001	[−0.002, 0.02]	0.76
Paternal RD → education → parents’ reading interests and habits → emergent literacy	−0.001	[−0.002, 0.01]	0.69
Maternal education → emergent literacy	0.05	[−0.08, 0.15]	0.39
Paternal education → emergent literacy	0.05	[−0.08, 0.15]	0.39
Access to print → emergent literacy	0.39	[0.01, 0.68]	0.01
Reading-related activities → emergent literacy	0.37	[0.02, 0.35]	0.01
Parents’ reading interests and habits → emergent literacy	0.26	[0.001, 0.15]	0.01

*Note.* CI = confidence interval; RD = reading disability.

A Wald test was applied to examine whether the direct and indirect pathways from maternal self-reported RD to code-related emergent literacy differed statistically from those of the paternal pathways. The results showed no significant differences, *χ*^2^(5) = 6.39, *p* = 0.27. This finding suggests that the associations between parents’ self-reported RD, the HLE and children’s code-related emergent literacy did not differ between the mothers and the fathers when the parents’ educational levels were accounted for.

## Discussion

First, this study supports a three-factor HLE model that includes *parents’ reading interests and habits*, *reading-related activities*, and *access to print*. Second, the results add to the literature by studying how the FR of RD is linked to each of these HLE domains while accounting for the significant role of the parents’ educational levels. Finally, the associations between the HLE, the parents’ educational levels, parents’ self-reported RD and children’s code-related emergent literacy were tested in a broader structural model. The results suggest that children’s code-related emergent literacy outcomes and their experiences in their home (HLE) may not be independent of risk factors such as FR of RD, even though the parents’ educational levels were accounted for. However, the HLE is a dynamic and modifiable environmental factor that could enhance children’s emergent literacy. These findings are discussed below.

### Three-Factor Home Literacy Environment Model

The three-factor HLE model suggested by this study extends the previous HLE research in two ways. First, the majority of the previous HLE research (Burgess et al., 2002; Hamilton et al., 2016, 2021; Sénéchal, 2006; Sénéchal et al., 1998; Sénéchal & LeFevre, 2002, 2014) has examined the domain of *exposure to print* as a single-factor model that included two distinct domains of the HLE (*access to print* and *reading-related activities*) together as one factor. In line with the distinction of the *passive* and *active* aspects of the HLE suggested by Burgess et al. (2002), this study established that *access to print* and *reading-related activities* should be considered two distinct factors.

Second, the domain of *parents’ reading interests and habits* was added to the HLE model as the third factor in this study. In line with Weigel et al. (2006), the present study found that the domain of *parents’ reading interests and habits* was strongly associated (0.73) with the domain of *access to print* and moderately associated (0.49) with the domain of *reading-related activities* (see Figure 1). These findings suggest that parents with high reading interest might provide more reading material at home, read to their children at an early age and read to them more frequently than those with low reading interest.

### Associations Between the Home Literacy Environment, Parents’ Self-Reported Reading Disabilities, and Parents’ Educational Levels

Model 2 (see Figure 2) shows that, when the parents’ educational levels were accounted for, maternal self-reported RD was directly and negatively associated with two domains of the HLE (i.e., *access to print* and *parents’ reading interests and habits*), but it was not associated with the domain of *reading-related activities*. However, the high maternal educational levels were positively associated with all three domains of the HLE when the negative association of maternal self-reported RD was accounted for. These findings suggest that mothers who self-report RD but have a high educational level might provide relatively good reading-related activities at home despite having reading problems and less reading interests and fewer reading habits of their own. This finding is somewhat in line with previous FR studies that found that *shared reading activities* and *access to print* did not differ between families with and without FR of RD when there were no differences between these groups in terms of either the parents’ (Torppa et al., 2007) or the maternal educational level (Elbro et al., 1998).

However, Model 2 shows that paternal self-reported RD was directly and negatively associated with two domains of the HLE (*access to print* and *reading-related activities*) but not with the domain of *parents’ reading interests and habits.* This finding suggests that the pattern of the association between maternal self-reported RD and the HLE might differ from that of the paternal association. However, the results of the Wald test indicate that the associations between maternal self-reported RD and the HLE domains when the parents’ educational levels were accounted for did not statistically differ from the paternal association pathways, even though Figure 2 suggests some patterns of differences.

A possible explanation for such patterns of differences could be related to the differences in the parents’ educational levels. In this study, the proportion of mothers with a high educational level was greater than the proportion of fathers with a high educational level in the whole sample as shown in Table 1, χ^2^ (1, *N* = 794) = 142.15, *p* < 0.001. The same pattern was found in the families both with and without FR of RD, indicating that regardless of whether the parents self-reported RD, the mothers reported higher educational levels than the fathers. Therefore, a higher level of maternal education might have eliminated the direct negative association between maternal self-reported RD and the HLE (the domain of *reading-related activities*) in this study. Therefore, the association between maternal self-reported RD and *reading-related activities* was fully accounted for by the maternal high educational level. This finding suggests that a high maternal education, as a protective environmental factor, might decrease the negative association between maternal self-reported RD and the HLE. However, the maternal educational level should not be considered a full environmental factor, as it is associated with maternal self-reported RD, which is a proxy for the FR of RD.

In conclusion, the findings obtained from Model 2 support the hypothesis that the parents’ educational levels play a significant role in the association between the HLE and the FR of RD (Dilnot et al., 2017; Esmaeeli et al., 2018; Hamilton et al., 2016), even though this association was not found to be fully mediated by the parents’ educational levels.

It is also worth mentioning that the parents’ educational levels were used as a proxy for family SES because investigating the contribution of the FR of RD and parents’ educational levels was the main interest in this model. In this study, a high maternal educational level may suggest high family income or at least good attitudes toward the HLE; however, the impact of family income on the HLE (e.g., access to print) remains to be discussed.

### Associations Between the Home Literacy Environment, Parents’ Self-Reported Reading Disabilities, Parents’ Educational Levels, and Children’s Preschool Code-Related Emergent Literacy

Unsurprisingly, maternal and paternal self-reported RD, which is a known risk factor, had direct and negative associations with children’s preschool code-related emergent literacy when the associations between the parents’ educational levels and the HLE were accounted for (see Figure 3). Taken together, these findings, in line with the previous FR research, highlight the fact that parents’ self-reported RD is a unique predictor of children’s preschool code-related emergent literacy and explain some additional variance that could not be accounted for by either the parents’ educational levels or the informal HLE, as assessed in this study.

Nevertheless, this study adds to the existing HLE literature (Hamilton et al., 2021; Khanolainen et al., 2020; Manolitsis et al., 2011, 2013; Niklas & Schneider, 2013; Salminen et al., 2021; Silinskas et al., 2020; Torppa et al., 2022) by showing that the positive association between the HLE and children’s preschool code-related emergent literacy exists even after accounting for the parents’ educational levels, the direct and negative association between parents’ self-reported RD and children’s code-related emergent literacy, and the indirect associations between parents’ self-reported RD and children’s code-related emergent literacy via the parents’ educational levels and the HLE. These findings, which are based on the multiple-deficit model of RD, suggest that the HLE plays a significant part in the complex relationships between children’s emergent literacy, the FR of RD and the parents’ educational levels.

In summary, FR of RD is a risk factor that put children of parents with self-reported RD to face a great risk of RD (Snowling & Melby-Lervåg, 2016). However, HLE as early intervention programs can enhance children’s emergent literacy and their subsequent literacy progress, especially in the context of RD. According to the meta-analysis conducted by Flack et al. (2018), the effectiveness of HLE programs is more reliant on *reading style* (e.g., *how* reading-related and reading activities are offered to children at home) than to *who* offers such activities. Thus, we can invest in parents’ involvement in home literacy programs if we provide them with proper resources and instructions. HLE programs that encourage parents’ involvement and provide them with proper instructions may improve emergent literacy and subsequent literacy progress, both generally and in the context of RD or the risk of RD.

### Limitations and Directions for Future Studies

Like all studies, the present study has some limitations. First, similar to most previous research, in this study, the HLE measure was based on parental reports, which may have led to social desirability response bias. However, given that the parents’ responses covered the entire spectrum from low to high rates of reading-related activities at home, such bias may not be of great concern in the present data. This HLE did not address a *formal* HLE (i.e., parents’ direct teaching to promote literacy) or meaning-related emergent literacy skills, as this study was interested in the association between an *informal* HLE and children’s code-related emergent literacy at the onset of formal reading instruction. Future research needs to shed light on the links between formal HLEs and children’s preschool emergent literacy, including both code- and meaning-related skills, especially in the RD context and for children with FR of RD. In addition, the HLE did not include items regarding “digital reading activities” because this aspect was not part of the previous research upon which this study was based. More importantly, the present study intended to investigate parents’ print-related reading activities as a role model for their children in terms of reading habits and a pleasure activity. However, some parents use digital reading activities for both pleasure and work.

Second, the proxy used for FR of RD in this study was parents’ self-report of RD and was based on only two questions. Although the validity of this simple but valuable self-report questionnaire has been examined previously (reference), a more comprehensive self-report questionnaire for parents is recommended in future research.

Finally, this study has a cross-sectional and correlational approach because the aim was to investigate the association between the HLE and children’s emergent literacy skills before the onset of formal reading instruction. Much more longitudinal, intervention and experimental research is needed to establish the complex relationships between FR of RD, the parents’ educational levels, the HLE and children’s emergent literacy outcomes and their later reading skills after the start of formal schooling.

### Conclusions and Implications for Practitioners and Researchers

Despite these limitations, the present study has several important implications. First, *parents’ reading interests and habits* were strongly associated with *access to print*, moderately associated with *reading-related activities* at home, and significantly associated with children’s preschool code-related emergent literacy. This finding highlights the importance of including *parents’ reading interests and habits* in the HLE measures in future research.

Second, children with FR of RD are at a great risk of RD (Snowling & Melby-Lervåg, 2016); however, an enhanced HLE contributes to children’s emergent literacy development, which may improve their future literacy progress. Therefore, it is critically important to continue to study the mechanisms of the interaction between FR of RD, the HLE, emergent literacy and later literacy skills in complex interactive models of RD, such as that examined in the present study.

Finally, although this study is correlational and its findings should be interpreted with caution, practitioners should be aware of potentially how having a history of RD within a family (FR of RD) is important to children’s emergent literacy outcomes and of how supporting parents may help to enhance children’s preschool code-related emergent literacy and later literacy outcomes via an enriched HLE. The complex, interactive model of RD used in this study indicates that the HLE is positively associated with children’s emergent literacy even after accounting for direct and indirect negative associations of the FR of RD and the parents’ educational levels. Based on this positive and relatively moderate association between the HLE and children’s emergent literacy, consistent with previous research, as well as the positive effect reported in previous research on family literacy programs (Fikrat-Wevers et al., 2021; Mol & Bus, 2011), the present study suggests that an enhanced HLE (e.g., a high-quality home literacy program such as that suggested by Flack et al., 2018) could operate as a potential protective environmental factor that might decrease the negative associations between the FR of RD and children’s emergent literacy. The weakening of these negative associations might in turn reduce the likelihood of emergent literacy difficulties in children with FR of RD, according to the multiple-deficit model of RD (van Bergen, van der Leij, & de Jong, 2014). In summary, the findings emphasize that home literacy activities, which might have a potential protective role in promoting children’s emergent literacy skills, should be added to any early literacy protection and intervention program, especially in the context of FR of RD. Although FR of RD may recur among family members, an enhanced HLE can be considered an effective early intervention program to help children’s emergent literacy and subsequent literacy development.

More importantly, the current study shows that practitioners should pay attention to all aspects of the HLE as an early prevention and/or intervention program:

(a) Parents’ own reading interests and habits, as a reading culture within a family, can be considered the starting point of reading-related activities at home (Weigel et al., 2006). Parents in general and specifically parents with RD or parents with low educational levels (Fikrat-Wevers et al., 2021), may need encouragement and to be reminded of the part they play as role models who enjoy reading for pleasure and appreciate reading as a hobby. This behavior may facilitate the establishment of a culture of reading habits at home. As a part of the home literacy program, parents should be encouraged to express a positive attitude toward reading to their children by showing them that they appreciate reading as a pleasurable hobby. Parents should be encouraged to communicate a positive attitude to their children by operating as role models and discussing this appreciation explicitly with the children.
(b) Children’s access to print, such as the number of children’s books in the household or access to libraries, is an important consideration. Practitioners could consider providing such access in their prevention/early intervention home literacy programs. de Bondt et al.’s (2020) meta-analysis on “book giveaway programs,” in which free books were provided for families with young children, revealed that the presence of more age-appropriate books in the home encouraged parents and their children to engage in more shared reading activities as part of a reading habit culture.
(c) Reading-related activities as an active HLE domain should also be included as the main part of prevention/early intervention literacy programs. Parents should be introduced to effective shared reading activities such as dialogic reading styles (Flack et al., 2018) and methods that aim to make shared book-reading an enjoyable time of interaction between the parents and their children. Some parents need to learn how shared reading and reading-related activities can be fun and enjoyable. Furthermore, how to have additional interactions with the text—such as engaging in text-related talk, using open-ended questions and picture-pointing methods to understand the new concepts/words and repeating the same story several times to assure the learning of new words/concepts—should be added to home literacy programs (Flack et al., 2018). Similarly, another meta-analysis study concluded that “book giveaway programs” are particularly effective when they include shared reading instruction for parents and offer several personnel–parent information sessions (de Bondt et al., 2020).

In addition, parents should be encouraged to start reading to their children at an early age. This is because children who are exposed to book-reading activities at an early age are more likely to maintain an interest in reading and to develop better emergent literacy, oral language and subsequent literacy skills (Mol & Bus, 2011).

## Supplemental Material

sj-docx-1-ldx-10.1177_00222194231195623 – Supplemental material for A Model of the Home Literacy Environment and Family Risk of Reading Difficulty in Relation to Children’s Preschool Emergent Literacy Supplemental material, sj-docx-1-ldx-10.1177_00222194231195623 for A Model of the Home Literacy Environment and Family Risk of Reading Difficulty in Relation to Children’s Preschool Emergent Literacy by Sara Esmaeeli in Journal of Learning Disabilities
