# Supplementary material for: A Model of the Home Literacy Environment and Family Risk of Reading Difficulty in Relation to Children’s Preschool Emergent Literacy
Source: J Learn Disabil. 2023 Sep 16;57(3):181–96. doi: 10.1177/00222194231195623 (PMC11044524; doi:10.1177/00222194231195623)
Supplement: sj-docx-1-ldx-10.1177_00222194231195623 – Supplemental material for A Model of the Home Literacy Environment and Family Risk of Reading Difficulty in Relation to Children’s Preschool Emergent Literacy [file sj-docx-1-ldx-10.1177_00222194231195623.docx]

**JOURNAL OF LEARNING DISABILITIES SUPPLEMENTAL FILE**

**Appendix. Home Literacy Environment Questionnaire**

**ARTICLE TITLE: A Model of the Home Literacy Environment and Family Risk of Reading Difficulty in Relation to Children’s Preschool Emergent Literacy**

- “How many children’s books/picture books do you have at home?”

(1 = None, 2 = 1-10 books, 3 = 11-20 books, 4 = 21-40 books, 5 = more than 40 books).

- “How often do you take your child to a public library?”

(1 = rarely or never, 2 = Rarely, 3 = about once a month, 4 = once a week 5 = almost several times a week).

- “How old was your child when you first started reading to her or him?”

(1 = We rarely/never read to our child, 2 = about 4-5 years old, 3 = about 3-4 years old, 4 = about 2-3 years old, 5 = before the age of two).

- “How often do you read to your child?”

(1 = rarely or never, 2 = about once a month, 3 = about once a week, 4 = almost several times a week, 5 = almost daily)

- “How often does your child watch TV or play games on a computer/tablet/mobile phone on a daily basis?”

(1 = more than three hours, 2 = about 2-3 hours, 3 = about 1-2 hours, 4 = about less than one hours, 5 = rarely or never).

- “How often do fathers/mothers read books or newspapers and magazines?”

(1 = rarely or never, 2 = about a few times a year, 3 = almost monthly, 4 = almost once a week, 5 = almost several times a week).

- “Please rate your level of agreement with this statement: (“*I only read if I have to*”)

(1 = completely agree, 2 = agree to some extent, 3 = disagree to some extent, 4 = completely disagree).
